# Epidemiological Shifts and Trends From 2009 to 2023 in Hospital Admissions for Ruptured Aortic Aneurysms and Aortic Dissections in Austria: Retrospective, Population-Wide Study

**DOI:** 10.2196/81702

**Published:** 2026-06-12

**Authors:** Fadi Taher, Maria Elisabeth Leinweber, Afshin Assadian, Amun Hofmann

**Affiliations:** 1 Department of Vascular and Endovascular Surgery, Klinik Ottakring, Montleartstraße 37, Vienna, 1160, Austria, 43 491504103

**Keywords:** aortic aneurysm, rupture, dissection, incidence, mortality

## Abstract

**Background:**

Aortic pathologies in general and acute aortic syndromes in particular are persistent public health concerns worldwide. Continuous efforts to monitor and update incidence rates are necessary for guided public health interventions and health care policy adaptation.

**Objective:**

This study aims to evaluate temporal patterns in the incidence and outcomes of hospital admissions for ruptured aortic aneurysms (rAAs) and aortic dissections (ADs) in Austria over a 15-year period, with a focus on population-wide trends.

**Methods:**

This study provides a retrospective, population-wide analysis of aortic syndromes using national health care data from 2009 to 2023. The data were provided by a federal data warehouse that stores all recorded in-hospital services in Austria. We analyzed annual and population-adjusted hospital admissions, in-hospital mortality, comorbidities, complications, and demographic characteristics for rAA and AD cases. Temporal patterns were assessed for annual variations. Exploratory forecasts were made until 2030 using exponential smoothing time series models. Subgroup analyses by sex and age strata were incorporated to contextualize epidemiological shifts and assess differential trends across population segments.

**Results:**

Corresponding to worldwide trends, the proportion of individuals aged 65 years and older slightly increased in Austria, from 17.4% in 2009 to 19.6% in 2023. Hospital admissions for rAA decreased from 357 in 2009 to 292 in 2023 (−18.2%), with admission rates falling from 4.3 to 3.2 per 100,000 (−25.2%) over the study period. In-hospital mortality among patients with rAA declined from 38.7% (138/357) to 34.6% (101/292), accompanied by a decrease in major comorbidities and recorded in-hospital complications. The incidence of hospital admissions for AD, however, showed a significant increase from 430 to 872 (+102.8%), with population-adjusted incidence rates rising from 5.2 to 9.6 per 100,000 (+85.6%). In-hospital mortality for aggregated AD cases decreased slightly, from 12.1% to 11.0%. Forecasting analyses suggest a continued rise in AD incidence through 2030, whereas rAA rates are expected to remain comparatively stable.

**Conclusions:**

While rAA incidence and mortality declined, AD cases showed a substantial increase, potentially due to improved detection, diagnostic advances, or a true epidemiological shift that warrants further investigation. These diverging trends highlight the need for updated surveillance strategies, improved risk stratification, and adaptation of health care resources to a potentially increasing burden of AD.

## Introduction

The worldwide prevalence of both thoracic and abdominal aortic aneurysms (AAAs) has risen over the past two decades, attributable largely to increased detection through screening and the aging of populations in low- and middle-income countries [1-4]. However, high-income countries have experienced a decline in prevalence, rupture rates, and aneurysm-related mortality from historic peaks, reflecting the success of screening programs, reduced smoking prevalence, and improved management of hypertension [3,5,6]. A recent meta-analysis of 54 population-based studies suggested an AAA prevalence of 1.75% in men and 0.57% in women in Europe, which is in line with contemporary screening studies from this region [3,7,8]. Alongside the reduction in non-ruptured AAAs, driven by changes in cardiovascular risk factors, broader use of preventive elective surgeries through screening programs, and the widespread adoption of endovascular aneurysm repair, the prevalence and mortality of ruptured aortic aneurysms (rAAs) have markedly declined [9,10]. Nonetheless, rAAs remain a relevant cause of vascular death, with pre-hospital mortality often exceeding 50% and reported in-hospital mortality rates of up to 25% even after successful surgical treatment [11-13]. The advent of endovascular aneurysm repair (EVAR) has markedly reduced mortality in patients with rAAs. Compared to open surgical repair, EVAR is associated with lower perioperative morbidity, reduced hospital stays, and improved short-term survival [9].

The epidemiology of aortic dissections (ADs) is well researched, with significant geographical and temporal variations in incidence. A population-based study in Olmsted County, Minnesota, from 1995 to 2015 reported an incidence of AD of 4.4 per 100,000 person-years, with men being more affected than women [14]. A systematic review and meta-analysis estimated a worldwide incidence of acute ADs of 4.8 per 100,000 individuals per year, with type A dissections occurring at 3.0 per 100,000 and type B dissections at 1.6 per 100,000 [2]. In contrast, a study in Miyazaki prefecture in Japan reported a much higher AD incidence of 17.6 per 100,000, likely due to an older population and comprehensive postmortem computed tomography analysis, which better captured pre-hospital deaths [15,16]. European studies have demonstrated variable incidence, with rates ranging from 2.9 per 100,000 in Hungary to 6 per 100,000 in the United Kingdom, while Sweden reported incidences between 3.3 and 7.2 per 100,000, depending on study methodology. Ontario in Canada reported an incidence of 4.6 per 100,000, whereas Medicare data in the United States indicated a higher hospitalization rate of 10 per 100,000. In Asia, estimates range from 2.8 per 100,000 in China to 10 per 100,000 in Tokyo, Japan [17]. Despite geographical differences, the high mortality associated with AD remains a significant concern, with a 30-day mortality rate as high as 57% in Japan [16].

This study provides a detailed temporal analysis of rAA and AD in the Austrian population over a 15-year observational period, leveraging *International Classification of Diseases, 10th Revision* (ICD-10)–coded health claims data. It thereby aims to investigate potentially contrasting trends in hospital admissions for rAA (ICD-10 codes I71.1, I71.3, I71.5, and I71.8) and AD (I71.0) and incidence, explore possible contributing factors, such as diagnostic advancements or a shifting risk profile of patients, and assess the impact of health care accessibility, including potential effects of the COVID-19 pandemic.

## Methods

### Data

Data included in this analysis are derived from a federal database that covers in-hospital medical services in Austria. The database in its current structure was established in 2009; therefore, data between 2009 and 2023 were extracted for analysis. ICD-10 codes were used to classify cases. rAAs were defined as I71.1, I71.3, I71.5, and I71.8; AD as I71.0; acute myocardial infarction as I21, stroke as I63, I64, and G45, respiratory complications as J00-J22, coronary heart disease as I25, diabetes mellitus as E10-E14, and pulmonary comorbidities (including chronic obstructive pulmonary disease, asthma, and chronic bronchitis) as J41-45. The current ICD-10 code I71.0 represents a composite code for all types of AD, encompassing types A and B as well as acute, subacute, and chronic presentations, which is a limitation inherent to ICD-10. Furthermore, the proportion of male and female patients was obtained, as well as age strata (60 years and younger, 60-69 years, 70‐79 years, and 80 years and older) and length of hospital stay strata (less than 9 days, 10-14 days, 15-19 days, and more than 19 days). These data were obtained as aggregated cases per year. Mortality refers to all-cause in-hospital mortality for cases with rAA or AD recorded as the main diagnosis. Additionally, daily case numbers and associated surgeries were provided for rAA and AD. All data were obtained by the Austrian Ministry of Health through the DIAG (Dokumentations- und Informationssysteme für Analysen im Gesundheitswesen) data warehouse. Diagnoses and services were recorded by health care professionals and administrative staff in hospitals and underwent quality control checks at multiple stages. Data covering population size (at the start of the calendar year) and smoking habits were extracted from the publicly accessible databases of Statistics Austria, the federal Austrian statistical office.

### Ethical Considerations

Requirement for institutional review board approval was waived by the ethics committee of the City of Vienna due to the exclusive use of anonymized, aggregated data.

### Descriptive Analysis

This study is primarily an ecological study investigating national aggregated data. Descriptive analysis included inspection of measures of central tendency and dispersion. A qualitative and quantitative analysis of temporal patterns was conducted, including yearly changes and changes across the observational period. Incidence rates of hospital admissions reflect the number of cases per 100,000 person years. Since all data are aggregated per year, this rate is equal to per 100,000 inhabitants.

### Forecasting Future Event Rates

To estimate future trends in the incidence of rAAs and ADs, we applied time series forecasting using exponential smoothing (Error, Trend, Seasonal [ETS]) models. Analyses were conducted separately for men and women, and rates were calculated as mentioned above. We generated univariate time series for each combination of event type (dissection or rupture) and sex. ETS models were fitted to each time series using the *forecast* package in R (version 4.4.2), which automatically selects the best-fitting model form (additive or multiplicative errors, trend, and seasonality) based on information criteria. Forecasts were projected from 2024 to 2030, including 95% CIs. This approach allowed us to estimate future sex-specific incidence trends under the assumption that past dynamics will continue.

## Results

### Population Characteristics

**Figure 1.**
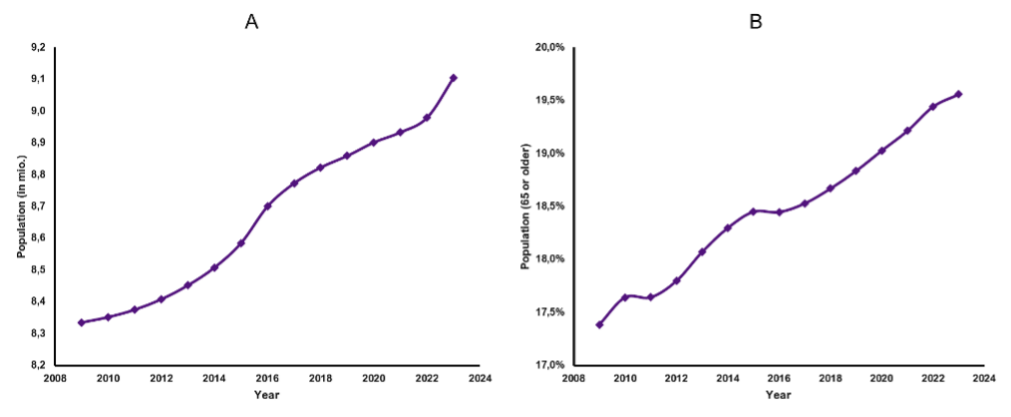
Demographics of the Austrian population from 2009 to 2023. (A) Population size (in millions). (B) Proportion of the population aged 65 years or older.

From 2009 to 2023 the Austrian population increased from 8.3 to 9.1 million inhabitants (Figure 1). The demographic structure remained rather stable over the observational period. The population consisted of 50% to 51% female inhabitants. Corresponding to worldwide trends, the proportion of individuals aged 65 years or older slightly increased, from 17.4% in 2009 to 19.6% in 2023. As smoking and arterial hypertension constitute major risk factors for both pathologies, we investigated the datasets of Statistics Austria. While yearly data on smoking were not available, Statistics Austria reported that for men and women, daily smoking decreased from 27.5% and 19.4% in 2006 to 23.7% and 17.9% in 2019, respectively. The prevalence of arterial hypertension is also documented by Statistics Austria and did not change substantially between 2006 and 2019, affecting around a fifth of the adult population. The proportion of men with hypertension slightly increased, from 20% in 2006 to 22% in 2019, whereas in women it decreased from 23% to 21.6% [18,19]. Table 1 illustrates the main metrics of interest.

**Table 1.** Main metrics of interest regarding trends in population size, ruptured aortic aneurysms (rAAs), and aortic dissections (ADs) in Austria between 2009 and 2023.

Metrics	Median (IQR) between 2009 and 2023	2009	2023	Relative change
Population size (millions)	8.7 (8.4-8.9)	8.3	9.1	9.2%
Admissions for rAA (n)	303 (291-313)	357	292	−18.2%
Admissions for AD (n)	687 (507-720)	430	872	102.8%
Admission rate for rAA (per 100,000)	3.5 (3.2-3.7)	4.3	3.2	−25.2%
Admission rate for AD (per 100,000)	7.8 (6.0-8.1)	5.2	9.6	85.6%
In-hospital mortality rate for rAA (%)	36.7 (34.2-40.0)	38.7	34.6	−10.6%
In-hospital mortality rate for AD (%)	12.1 (11.7-13.1)	12.1	11.0	−9.0%

### Trends in rAAs

**Figure 2.**
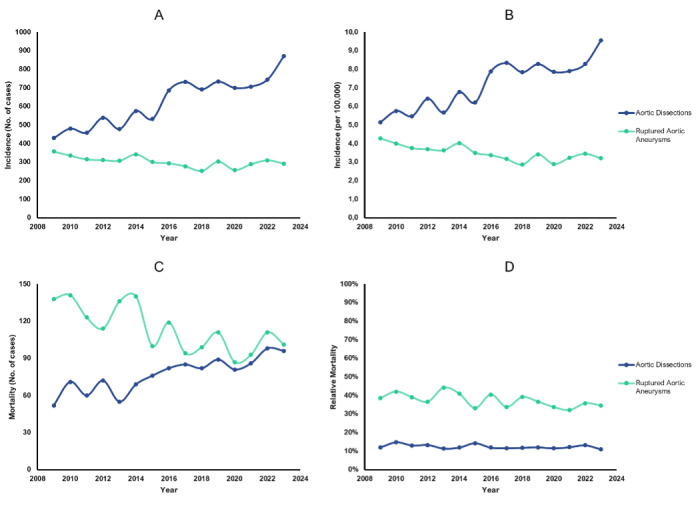
Trends in ruptured aortic aneurysms and aortic dissections in Austria from 2009 to 2023. (A) Hospital admissions in absolute case numbers. (B) Admission rate per 100,000 for ruptured aortic aneurysms. (C) Mortality (absolute). (D) Mortality as a proportion of all cases (as a percentage).

**Figure 3.**
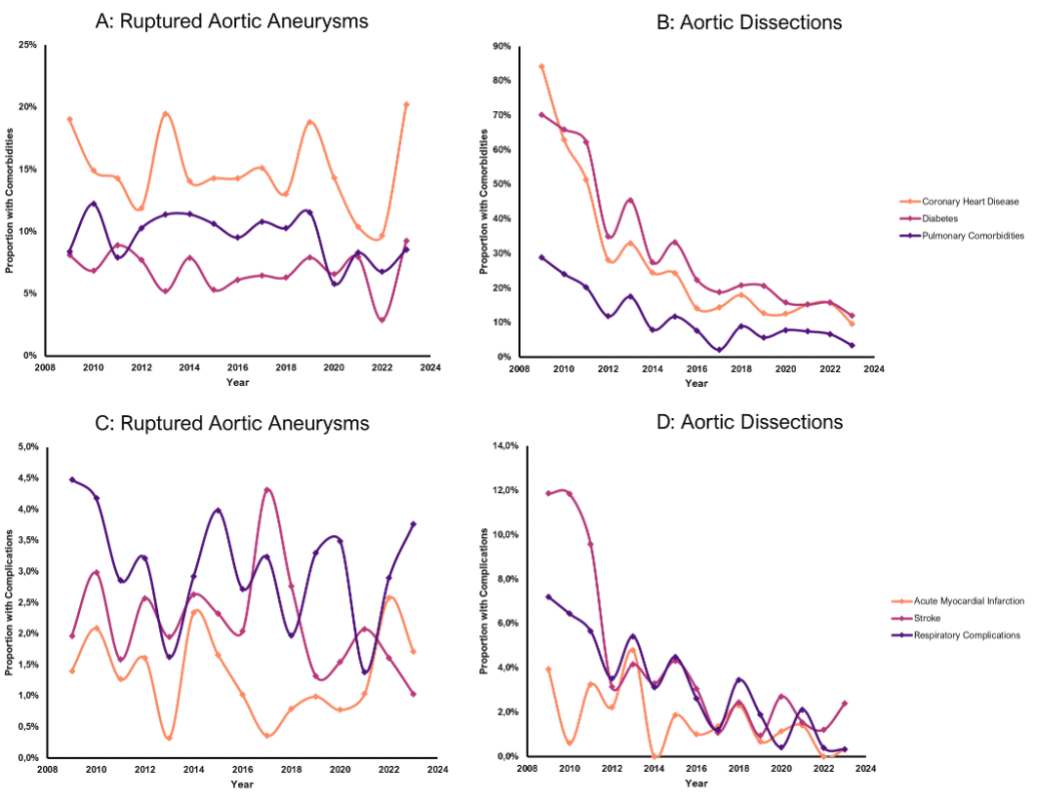
Comorbidities and complications in cases of ruptured aortic aneurysms and aortic dissections in Austria between 2009 and 2023. (A) Proportion of ruptured aortic aneurysm cases with coronary heart disease, diabetes, or pulmonary comorbidities (B) Proportion of aortic dissection cases with coronary heart disease, diabetes, or pulmonary comorbidities (C) Proportion of ruptured aortic aneurysm cases with recorded in-hospital complications (D) Proportion of aortic dissection cases with recorded in-hospital complications.

Hospital admissions for rAAs declined from 357 cases in 2009 to 292 cases in 2023 (–18.2%) during the observational period (Figure 2A). Adjusting for the increase in population size, admission rates fell from 4.3 to 3.2 per 100,000 (–25.2%; Figure 2B). Cases with a recorded in-hospital death decreased over the study period (Figure 2C). This corresponds to a relative in-hospital mortality decrease from 38.7% (138/357) of all cases in 2009 to 34.6% (101/292) in 2023, even though this was not a monotonic development, with a peak of 44.2% (136/308) in 2014 (Figure 2D). Of note, this was also accompanied by a decrease in major comorbidities (Figure 3A) and recorded in-hospital complications (Figure 3C).

There were no relevant changes in length of hospital stay during the observational period, with a median of 61.6% (range 56.1%-65.4%) of patients having a recorded length of stay of 9 days or less. Age structure also remained rather stable, with a median of 15.9% (range 12.6%-21.2%) of patients being aged 59 years or younger, a median of 21.3% (range 16.9%-26.0%) aged 60 to 69 years, a median of 31.3% (range 22.1%-36.8%) aged 70 to 79 years, and 30.3% (range 26.5%-39.8%) aged 80 years or older. Sex distribution of cases also remained stable, with a median of 29.5% (range 26.0%-38.0%) of rAAs occurring in female patients and correspondingly a median of 70.5% (range 62.0%-74.0%) in male patients.

### Trends in ADs

Hospital admissions for ADs increased from 430 cases in 2009 to 872 in 2023 (+102.8%). The incidence rate rose from 5.2 to 9.6 per 100,000 (+85.6%; Figure 2A and B). Recorded in-hospital mortality decreased slightly from 12.1% to 11.0% over the study period (Figure 2C and D). In the early years of the observational period (2009‐2013), AD cases were more frequently associated with cardiovascular and pulmonary comorbidities than in later years (Figure 3B and D).

A median of 39.1% (range 34.5%‐44.5%) of patients were aged 59 years or younger, a median of 26.6% (range 19.3%‐31.2%) were aged 60 to 69 years, a median of 23.5% (range 19.1%‐29.0%) were aged 70 to 79 years, and a median of 10.2% (range 7.2%‐13.0%) were aged 80 years or older. A median of 35.6% (range 28.1%‐37.2%) of affected patients over the observational period were female and correspondingly a median of 64.4% (range 26.0%‐62.8%) were male.

### Surgical Treatment of Aorta Pathologies

**Figure 4.**
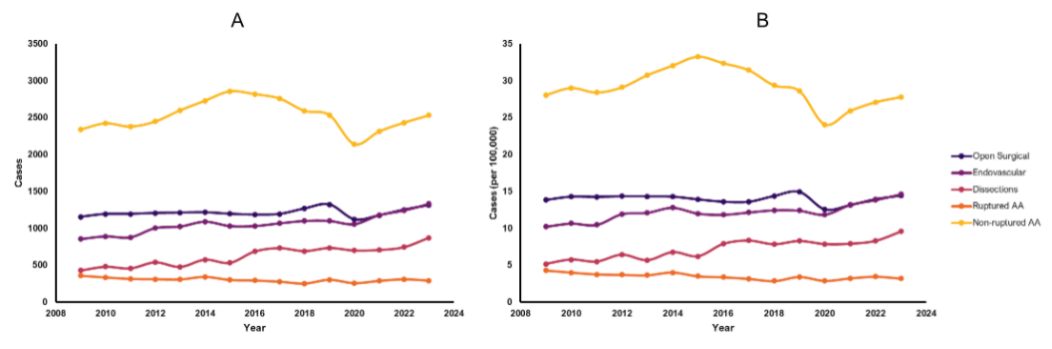
Temporal patterns regarding surgical treatments in Austria between 2009 and 2023. (A) Aortic surgeries (absolute). (B) Aortic surgeries per 100,000. AA: aortic aneurysm.

Figure 4A and B illustrate the temporal pattern of aortic surgeries in total and per 100,000 in Austria from 2009 to 2023. To explore a potential relationship between elective invasive treatments for nonruptured aortic aneurysms and the incidence of rAA, we investigated treatment patterns in elective settings. Elective treatments peaked in 2015 and steadily declined after that. With the start of the COVID-19 pandemic in 2020, both nonruptured aortic aneurysm admissions as well as surgical aortic aneurysm treatments showed a local minimum and a recovery of the caseload in the following years, returning to previous patterns. Endovascular aortic surgeries did not exhibit a corresponding trend, as they showed the steepest increase following the pandemic and a gradual increase over the study period. AD cases also showed a rather continual increase throughout the observational period. While no absolute increase in rAA cases was noted after the pandemic, the steepest increase in rAA cases did occur in the years after 2020.

### Midterm Forecasts

**Figure 5.**
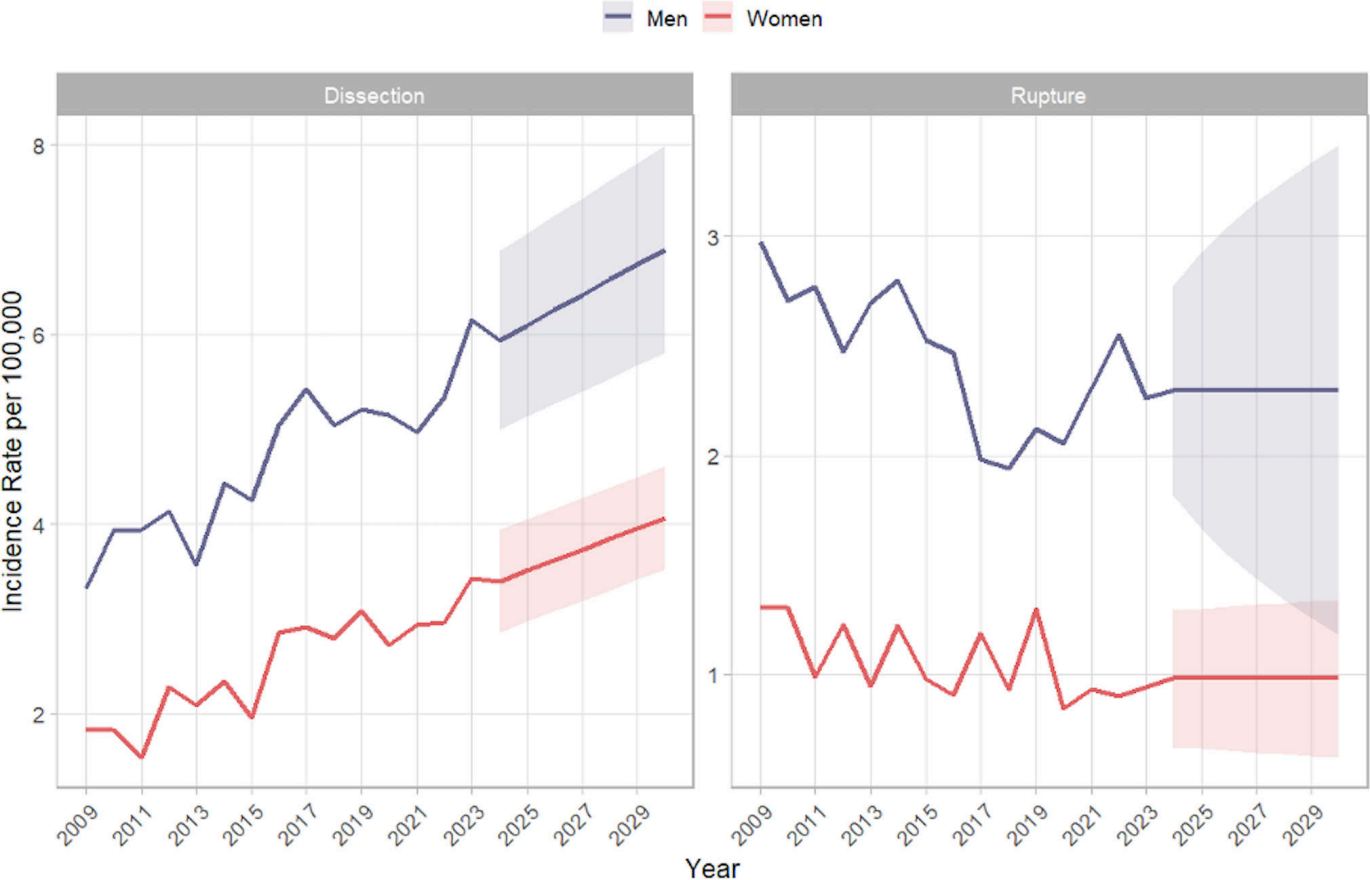
Forecasted incidence rates of aortic dissection and aortic rupture by sex from 2009 to 2030. Solid lines represent historical rates per 100,000 population from 2009 to 2023, calculated separately for men and women. Shaded ribbons indicate 95% CIs of the forecasted rates for 2024 to 2030, derived using exponential smoothing time series models.

In an exploratory analysis, forecasts for AD and rAA cases were made from 2024 through 2030, leveraging ETS models (see Methods). Incidence rate forecasts for AD cases among both male and female patients continuously increased, with a peak of 6.9 (95% CI 5.8‐8.0) in male patients and 4.1 (95% CI 3.5‐4.6) in female patients. This would result in a doubling of population-adjusted AD cases in about 20 years. Regarding rAA cases, estimated forecasts remained constant to the observational period from 2009 to 2023 in both sexes. These forecasts are illustrated in Figure 5.

## Discussion

This population-wide analysis highlights relevant changes in hospital admissions and outcomes of rAA and AD in a growing Austrian population over a 15-year period. The observed decline in rAA-related admissions and mortality contrasts a marked increase in AD cases, warranting further investigation into potential contributing factors. The admission rate for rAA decreased by 25.2% between 2009 and 2023, while the rate of AD increased by 85.6% over the same period. In-hospital mortality decreased slightly for both conditions, with relative reductions of around 10% over the observational period. The epidemiological profiles of affected patients correspond well with previous literature, with rAA occurring more often in older patient groups, whereas AD was frequently observed in patients younger than 60 years. Both pathologies affected male patients almost twice as often as female patients [20,21].

Epidemiological shifts in aortic pathologies are accompanied by corresponding trends for associated risk factors. Smoking, a main contributor to aortic aneurysm occurrence and rupture, has declined in many high-income countries in recent decades. The World Health Organization (WHO) estimates that tobacco use prevalence has more than halved between 2000 and 2025 in Austria, a trend that can be observed in other European countries, such as Germany or the United Kingdom, to a comparable extent. During the same period, the WHO has reported a decrease in the prevalence of hypertension among many European countries, even though estimates commonly show reductions of 20% to 25% [22]. Nevertheless, it should be considered that the point prevalence of a pathology is merely one aspect, with disease management being at least equally important to understand the epidemiological burden. As for arterial hypertension, advancements in treatment strategies and patient care have resulted in vastly improved blood pressure control in high-income countries [23,24]. The same considerations should be applied when interpreting the present data.

Reports of increased occurrence of AD from different geographical regions are not uncommon in the current literature [14]. The rise may be attributed to a variety of different variables, which may include improved diagnostics and therapeutic advances [25] that allow invasive treatment in historically inoperable cases. Developments in imaging as well as availability and use of high-resolution imaging in Austria may also have led to more frequent detection of AD, especially in the earlier stages, akin to how screenings can temporarily increase incidence rates. Because of this, the critical question remains whether the rise reflects a true increase in disease occurrence or an artifact of improved case ascertainment. A possible explanation could therefore be an increase in elective cases that undergo invasive treatment. Nevertheless, the absence of information regarding urgency and morphology in ICD-10 codes for AD (I71.0) remains a challenge when analyzing health claims data and routine electronic health records. Literature estimates of AD mortality vary widely, being heavily influenced by dissection type, patient characteristics, treatment indications, and modalities. Type A dissections have a reported 30-day all-cause mortality of 16.9% to 22% with surgical intervention and up to 50% to 69% without it [26-28]. In contrast, type B dissections, managed medically or with endovascular repair, have lower mortality, typically 4.5% to 13.8% [28,29]. Patient factors such as age, comorbidities, and genetic predispositions further modulate outcomes, as do treatment settings (acute vs elective) and modalities (open surgery vs endovascular). The current study’s aggregation of all AD cases, encompassing types A and B, acute and elective presentations, averaged mortality estimates, as the lower mortality of elective or type B cases may offset the higher lethality of acute type A dissections. Moreover, the exclusion of pre-hospital deaths introduced a significant survival bias, as patients who die before reaching the hospital were not captured. Nevertheless, considering this amalgamation and the reporting of in-hospital mortality rather than 30-day mortality, the present data correspond well to comparable investigations [30].

Regarding rAAs, there might be less scope for interpretation of the present data in detail. A decrease in hospital admissions for AAAs in England and a shift toward endovascular treatment has recently been reported [31]. While reductions in the incidence of rAA were already observed a decade ago [10], the underlying cause, although debated, was considered to likely be multifactorial and at least partially attributable to the implementation of screening programs and subsequent increases in elective repair [32]. The present data correspond to this hypothesis, with increasing elective cases until 2015. Although Austria does not have an established national AAA screening program, we hypothesize that the increased use of EVAR and greater awareness among primary care physicians may have contributed to a concomitant and subsequent decrease in the incidence of rAA. The absence of a national screening program is primarily a policy decision by the federal government and the association of national health insurance institutions and stands in contrast to current clinical practice guidelines [9]. Small decreases and an implied negative trend in mortality over the observational period are also likely multifactorial and certainly require further investigation. However, the fluctuating temporal pattern and overall high rates indicate that ruptures—when they occur—are still a major hazardous cardiovascular event.

Invasive treatment for nonruptured aortic aneurysms in Austria increased continuously from 2009 to 2015, when they peaked, with a subsequent steady decline. However, during the height of the global COVID-19 pandemic, a noticeable decrease was observed, with a recovery in 2023 to prepandemic caseloads. The fact that the COVID-19 pandemic led to delayed access to health care services was a phenomenon observed worldwide [33,34] that affected many pathologies, including cancer [35], stroke [36], and acute myocardial infarction [37]. Despite the challenges the COVID-19 pandemic presented to health care systems worldwide, its effects on aortic aneurysm treatment and associated outcomes were reported to be limited from a public health perspective [38-40]. Nevertheless, it was previously hypothesized that decreases in elective aortic aneurysm repair during the pandemic could have caused an accumulation of untreated aneurysms and an increased risk for rAA [41]. The slight decrease in elective aortic aneurysm treatments in 2020 was accompanied by small increases in ruptures in 2021 and 2022. However, this trend appeared to reverse in 2023, and nonruptured aortic aneurysm treatments were already in decline from 2015 onward. During the same time, both hospital admissions with rAA as well as associated mortality declined. While effects of the COVID-19 pandemic on the treatment of aortic aneurysms in Austria cannot be completely excluded, they appear rather limited for the time being.

The forecasts presented in this study should be interpreted as exploratory and illustrative rather than definitive predictions. Nevertheless, the estimated continuous increase in AD cases could require adaptation of infrastructure and policies, as forecasts indicate a potential doubling of caseloads in 2030 compared to 2009. However, while ETS models provide a data-driven approach to estimate potential future trends in the incidence of ADs and ruptures, these projections are inherently uncertain and depend on the assumption that past patterns will continue unchanged. Consequently, these estimates should not be used as the sole basis for health care policy or resource allocation decisions. Instead, they should be seen as serving to provide an informed understanding of possible developments that may warrant attention. It is also essential to reassess and update these forecasts as new data become available, particularly because the most recent data included extend only through 2023, and additional information from subsequent years may significantly influence future trends and model accuracy.

The observational period, spanning 15 years, may not have fully captured longer-term trends, and more extensive time series analyses will be required in the future. Additionally, as the analysis was based on data from Austria, generalizing the findings to other countries or regions with differing health care systems, demographics, and lifestyle factors may be challenging. Furthermore, difficulties arise with the recording of ADs via ICD-10, since the code I71.0 condenses both acute and elective clinical presentations as well as variations in morphology, that is, type A, B, non-A, or non-B. Similarly, most rAA cases were coded as I71.8 (rupture of unspecified location), which prevented further analysis of affected anatomic segments. Potential unmeasured confounders, such as changes in diagnostic practices, population health behaviors, or health care access, could have influenced the observed trends. Moreover, limited data on smoking prevalence and other individual-level risk factors precluded a more granular analysis of their impact on AD and rAA. The available information on smoking trends restricts interpretations to broader temporal patterns rather than direct year-to-year associations with incidence changes, as annual fluctuations in smoking cannot be precisely correlated with yearly variations in rAA or AD rates. These limitations highlight the need for multicountry analyses and screening studies with more detailed individual-level data to corroborate and expand upon these findings. Furthermore, the included data are based on recorded in-hospital diagnoses and services, and misclassifications in claims data are a well-described limitation.

The database used here included and represented more than 99% of all in-hospital services in Austria, thereby truly enabling a nationwide evaluation of hospital admissions, practice patterns, and outcomes of aortic syndromes between 2009 and 2023. However, limitations of the available data within the database highlight the value of nationwide registries such as the Vascular Registry in Sweden. Nevertheless, the present analysis offers insights into the evolving patterns of aortic pathologies, which may deserve consideration in health care planning and patient management, while adding recent data to the cumulative body of evidence on aortic pathologies.

In light of shifting demographics, changing comorbidity profiles, and evolving diagnostic and treatment options, aortic syndromes continue to be relevant from a public health perspective.

## Abbreviations

AAA: abdominal aortic aneurysm
AD: aortic dissection
EVAR: endovascular aneurysm repair
ICD-10: *International Classification of Diseases, 10th Revision*
rAA: ruptured aortic aneurysm
WHO: World Health Organization
